# Trichinosis

**Published:** 1881-06

**Authors:** 


Trichinosis.—Professor Rand of Philadelphia suggests in the *College and Clinical Record*, the use of Mercury and sulphur in the treatment of trichinosis on theoretical grounds. The former article he advises either by the mouth, inunction, or fumigation, until ptyalism is induced. Or the use of sulphur, in a similar manner, we presume. Both these articles are known as the most potent destroyers of the low forms of organisms, and we think the suggestion of their use is, to say the least, rational, and ought to be acted upon for destroying the trichina spiralis, when found in the human body.

